# A comprehensive evaluation of physical activity on sidewalks and streets in three U.S. Cities

**DOI:** 10.1016/j.pmedr.2022.101696

**Published:** 2022-01-19

**Authors:** Richard R. Suminski, Gregory M. Dominick

**Affiliations:** The Center for Innovative Health Research and the Department of Behavioral Health and Nutrition, University of Delaware, Newark, DE 19716, United States

**Keywords:** PA, physical activity, WVD, wearable video device, Health behavior, Environment, Observation method, Public health, Measurement

## Abstract

A considerable proportion of outdoor physical activity (PA) is done on sidewalks/streets. The purpose of the current study was to create a comprehensive picture of PA and non-PA (sitting and standing) occurring on sidewalks/streets. A wearable video device was used to capture videos during 2019 in three different size (small, medium, large), U.S. cities along 24 observation routes (sidewalks/streets) located in 24 study areas that varied in walkability, income level, and minority composition. Videos were collected over the course of one year during different times of week and weekend days. Expert reviewers examined each video to extract data on counts of people engaged in different types of activities (e.g., walking) per minute of video. A total of 1154 individuals were described in 1237 min of video as either walking (66.9%), sitting/standing (25.7%), jogging (4.2%), cycling (1.8%) or skating/playing (1.4%). A greater number of active people/min were seen in the evening and in the small city (*P* < 0.05) while more non-PA people/min were observed in the medium city and during the weekend (P < 0.05). Active and non-PA people/min were associated with walkability, income, and minority composition. For instance, in high walkability, low income areas, 3.2 active people/min were observed in areas with a high percentage of non-minority residents compared to 0.9 active people /min in areas with a high percentage of minority residents. Sidewalks/street activities are related to dynamic interactions between social and physical environmental factors. The results of this study may serve as a reference to which future, similar evaluations can compare.

## Highlights

• Video-enhanced observation provides objective data on sidewalks/streets activity.
• Neighborhood-level walkability was strongly associated with sidewalks/streets use.
• Most people (67%) were walking; others were sitting, standing, cycling, jogging.
• Sidewalk/street activity varied by time of day, weather, & environmental condition.

## 1 Introduction

It is well known that physical activity (PA) improves health and wellbeing and that physically inactive individuals are at increased risk of developing and dying from chronic diseases such as type 2 diabetes, cardiovascular disease, and cancer (National Center for Health Statistics (US). Health, United States, 2019, National Center for Health Statistics (US). Health, United States, 2017, Ward et al., 2015). Federal, state, and local efforts to promote PA have been ongoing for years, but are arguably not producing the desired impact. Recent national estimates indicated that 46.7% of U.S. adults do not meet guidelines for aerobic PA and 67% do not meet guidelines for both aerobic and muscle-strengthening activity (Clarke et al., 2019). Patterns of low PA are also seen in children especially those between 12 and 19 years of age (Alliance, 2018). Clearly, our understanding of PA and factors that influence it, needs enhancement to more effectively encourage PA engagement by individuals throughout the lifespan.

Interventions focusing on individuals are effective at promoting physical activity, but fall short of adequately addressing the robustness of the physical inactivity problem (Dishman, 1994, Dishman et al., 1994). In the past 15 years, there has been an exponential rise in studies looking at environmental (social and physical) determinants of health that affect PA, especially those associated with the built environment, collectively referred to as “walkability” (Benton et al., 2016 Oct 7, Brownson et al., 2009, Sallis et al., 2015). Neighborhood walkability components (e.g., mixed-land use, access to destinations) favorably influence the degree to which individuals engage in PA (Knuiman et al., 2014, Van Holle et al., 2012, Wilson and Cope, 2011, De Bourdeaudhuij et al., 2003, Salvo et al., 2015, Wendel-Vos et al., 2007). Sidewalks/streets are key components of walkability. They are common behavior settings for outdoor PA, particularly moderate-to-vigorous PA performed near one’s home. (Giles-Corti and Donovan, 2002, Kang et al., 2017, McCormack, 2017, Hurvitz et al., 2014, Suminski et al., 2015). It has been reported that nearly 70% of adults use sidewalks/streets in their neighborhood for recreational activity (Suminski et al., 2015). However, built environment variations do exist between neighborhoods producing inequities in walkability and subsequently lower rates of recreational activity on sidewalks/streets (Kelly et al., 2014). Sidewalks/streets take on added importance given they are essential aspects of transportation systems across the U.S. and around the world. For instance, in New York City there are 12,750 miles of sidewalks, 10,750 miles in Los Angeles, and 4,000 miles in Toronto, Canada (League of American Bicyclists, 2018).

Although a considerable amount of PA has been reported to occur on sidewalks/streets, few studies have actually observed PA occurring on sidewalks/streets, and those that have limited data collection to small, homogeneous geographical areas potentially restricting ranges of social and physical environmental factors (Suminski et al., 2015, Kelly et al., 2014, Dixon et al., 2021, Suminski et al., 2008, Suminski et al., 2008, Suminski et al., 2006). Information of this caliper would be useful for informing behavioral theories and future evaluations in this area. Therefore, the current study was conducted to provide the most comprehensive objective, description to date of sidewalk/street usage by humans for PA. A robust study design was adopted along with a widely used, observation method that has been shown to be most accurate when combined with advanced video capture techniques (McKenzie et al., 2002, Suminski et al., 2020, Suminski et al., 2019).

## 2 Methods

### 2.1 Study areas (n = 24)

Study areas were defined by U.S. Census block group boundaries and located within three cities of varying populations (19,928, 71,817, and 1,561,000 residents). City size was considered because it is associated a wide range of conditions (e.g., education, occupation type, population density, types of destinations) that could influence how humans use sidewalks/streets (Thomas et al., 2015). A description of the study areas is provided in Table 1.

**Table 1:** Characteristics of Study Areas.

	West Chester, PA (8 areas)	Wilmington, DE (8 areas)	Philadelphia, PA (8 areas)
Population	1300.3 (702.9)	899.1 (340.4)	1035.8 (420.2)
Population density (residents/square mile)	12,412.8 (6834.5)	8909.9 (7349.9)	10,313.3 (10,556.0)
Walk to work %	12.3 (6.0)	5.1 (4.9)	8.6 (13.7)
Cycle to work %	0.2 (0.5)	2.6 (7.4)	0.6 (0.8)
WalkScore	64.4 (28.9)	55.6 (29.5)	54.1 (37.2)
Median household income	51,048 (24,769)	63,011 (47,845)	54,404 (12,144)
Percent Minority	12.4 (10.9)	47.3 (37.1)	43.9 (40.0)

All values except total population are means +/- standard deviations for study areas; PA- Pennsylvania, DE – Delaware.

Policy Map was accessed for block group data on percentage of the residents who self-reported race and median household income (U.S. Census block group estimates for race and median household income, 2020). Minority composition was defined as the percentage of residents declaring their race as Black/African American, Asian, two or more races, or some other race. These racial categories accounted for over 99% of the racial groups in the study areas. Non-minorities were those indicating they were White. WalkScores were used as indicators of walkability. In brief, WalkScore combines publicly available data (e.g., distance to parks, stores) with an algorithm to generate scores ranging from 0 to 100 with 100 being the most walkable (Management, 2011). A study area’s walkability was based on the average WalkScore from 10 randomly selected addresses drawn from a list of all addresses in a given study areas. Previous research has shown WalkScore valid for estimating walkability and that it is significantly correlated with minutes/week of transport and leisure walking (Carr et al., 2011 Nov, Duncan et al., 2011, Hirsch et al., 2013).

Eight permutations of study areas were derived using the median values for WalkScore (<61.5 = low walkability), median household income (<46,448 = low income), and minority composition (<14.3% = low minority).

### 2.2 Observation routes

**Fig. 1:**
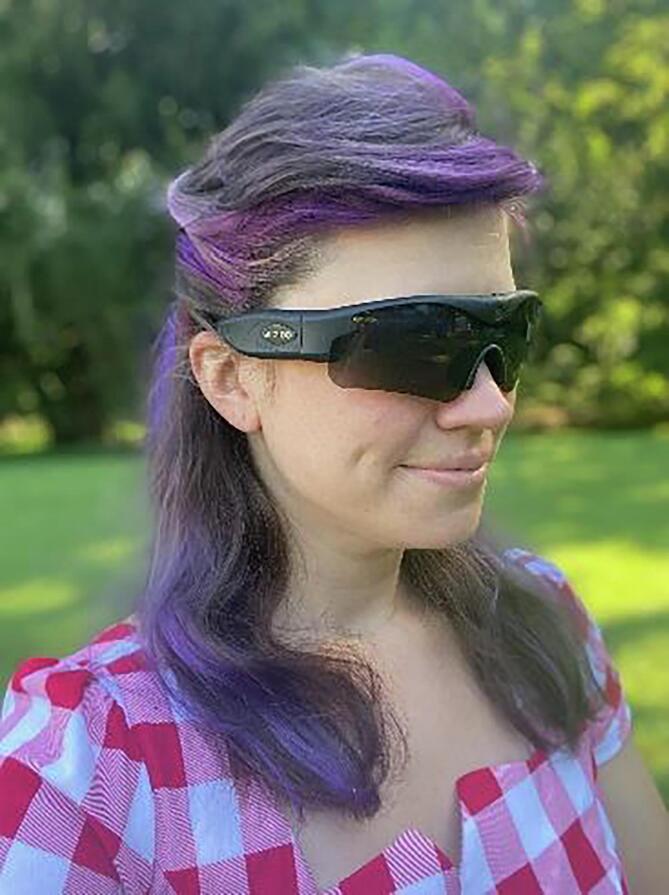
Gogloo E7 SMART WVD. (Written informed consent was obtained from the Individual for the publication of this image).

**Fig. 2:**
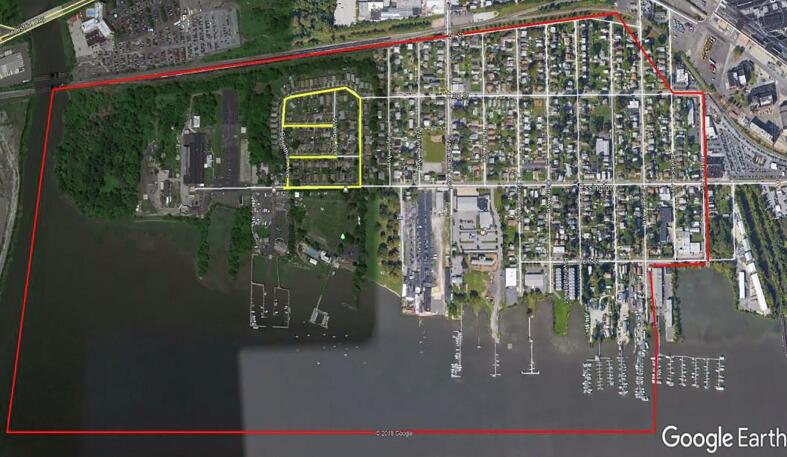
Example observation route highlighted in yellow (study area outlined in red).

The total linear length of sidewalks/streets in the study areas was estimated using the ruler tool in Google Earth, which is accurate to within + 1.5% for measuring street lengths (Suminski et al., 2006, Suminski et al., 2019). The total linear feet/study area was divided into 100 foot segments. One of the segments was randomly selected as the starting point with the remainder of the route extending continuously from this point until about 20% of the total linear feet within the block group was included. Positions along the route on both sides of the street were identified to serve as waypoints from where videos were captured with a wearable video device (WVD) (Gogloo E7 SMART eyewear, item model number E7B0100, www.goglootech.com) (Fig. 1). Maps of each route were constructed and the waypoint geo-coordinates (longitude and latitude) were included on the map (Fig. 2).

### 2.3 Meteorological conditions

Data on meteorological conditions (rainfall, relative humidity, temperature, wind speed, and barometric pressure) for the exact time of day observations were made, were obtained from an Automated Weather Sensor System (AWSS) located at a local airport.

### 2.4 Observation procedure

Each observation route was observed on a weekday and a weekend day between 8 and 9 a.m., 12–1p.m., and 5–6p.m., which is consistent with a previous study validating the observation method employed currently (Suminski et al., 2019). Observations were not conducted on days having an event that would affect counts (e.g., parade, marathon) or during times when it was precipitating. An observer, wearing the WVD, found each waypoint along the route using the Google maps app on their cellphone or referring to a pre-printed map. At each waypoint, the observer stood completely still while recording a 30 s video of the observation field in front of them and, once completed, walked the 100 feet to the next waypoint. If obstructions were encountered at a waypoint (e.g., parking meter), a new waypoint was established just beyond the obstruction. The University’s Protection of Human Subjects Committee judged this study exempt according to the Code of Federal Regulations issued by the Department of Health and Human Services.

### 2.5 Manual video Analysis

All videos were independently evaluated by two reviewers to determine numbers of people engaged in various activities. Detection boxes were displayed on each video to demarcate the area a person needed to be in order to count. The boxes stretched from the middle of the street to the inner most point of the sidewalk where the observer was positioned. All observation procedures and the extraction of data from videos are reliable and valid for counting people and describing their behaviors (Suminski et al., 2006, Suminski et al., 2020, Suminski et al., 2019).

### 2.6 Observer and video reviewer training

Two observers and two video reviewers participated in two training sessions before data collection. They were given detailed instructions on procedures for capturing video and extracting data from videos followed by mock field observations/video reviews.

### 2.7 Statistical Analysis

Pearson Product Moment correlation were derived to examine relationships between variables. Differences in video length between subgroups were explored using Student’s t-tests for dichotomous subgroup and Analysis of Variance (ANOVA) followed by Games-Howell (unequal variances) or Scheffe (equal variances) post-hoc tests for trichotomous subgroups. Analysis of Co-Variance (ANCOVA) was used to compare two outcome variables, active and non-active people seen/min of video (active/min and non-active/min) between observation periods, cities, and weekday vs. weekend days while controlling for meteorological conditions. Active included individuals physically active and non-active included individuals sitting or standing. Video length and meteorological conditions were controlled for due to their potential to confound relationships of interest. The ANCOVA procedure also was employed to explore differences in the outcome variables between study area permutations, while controlling for meteorological conditions. If an interaction effect was detected, difference in simple effects were examined using estimated marginal means (EMM) +/- Standard Error (SE) with Bonferroni adjustment for multiple comparisons. The significance level was set a priori at *P* < .05 and analyses were performed with IBM SPSS Statistics for Windows (Corp and Released, 2020).

## 3 Results

A total of 4470 videos amounting to 1237 min (27.7 +/- 0.1 s video/waypoint) were obtained along 90,587 linear feet of sidewalks/streets (3,774.0 +/- 1,614.0 linear feet/study area) during 10 different months in 2019 (January and June excluded). Video length/waypoint differed between observation periods (F(2,4469) = 3.2; *P* = .04), cities (F(2,4469) = 139.6; *P* < .001), weekday vs. weekend (t = 2.7; *P* = .007) and minority vs. non-minority areas (t = -5.5; *P* < .001). Video length did not differ between walkability categories (t = 0.60; *P* = .55) or income levels (t = 0.54; *P* = .59).

A wide range of meteorological conditions were noted: temperature (70.8 +/- 14.3; range 41-92°F), relative humidity (63.4 +/- 16.2; range 19–94%), wind speed (7.5 +/- 4.2; range 0–21 mph) and barometric pressure (29.8 +/- 0.4; range 29.1–30.5 mmHg). No precipitation fell on days data were collected. Humidity (r = −0.04; *P* = .005) and wind speed (r = -0.04; *P* = .02) correlated with active/min and temperature (r = 0.09; *P* < .001), humidity (r = -0.05; *P* = .002), and barometric pressure (r = 0.05; *P* < .001) correlated with non-active/min. All meteorological conditions differed across observation periods [F(2,4469) > 6.2; *P* < .003], cities [F(2,4469) > 602.9; *P* < .001], weekday vs. weekend (t > 9.0; *P* < .001), walkability category (t > -2.2; *P* < .03), income level (t > -3.0; *P* < .004), and minority category (t > 2.5; *P* < .02) (*note: only the lowest, significant statistics are presented for the relationships between meteorological conditions and subgroups*).

During the 1237 min of video footage, 1154 individuals were observed, of which, most were walking (including dog walking) (66.9%) and sitting/standing (25.7%). Few people were jogging (4.2%), cycling (1.8%) or engaging in any other types of activities. Walkers accounted for 90.1% of the 857 individuals who were physically active (Table 2).

**Table 2:** Number of people observed on sidewalks/streets by activity type.

	**Sum**	**% of Total**
Walking	733	63.5
Sitting/standing	297	25.7
Jogging	21	1.8
Cycling	48	4.2
Skate boarding	3	0.3
Roller blading	0	0
Playing	13	1.1
Dog walking	39	3.4
Total	1,154	

### 3.1 Comparative analysis active/min (controlling for meteorological conditions)

Active/min differed across observation periods with fewer seen during the morning compared to the afternoon and evening [F(2,4463) = 12.6; *P* < .001]. Variations between cities were noted with more active/min seen in West Chester (small) than in Wilmington (medium) and Philadelphia (large) [F(2,4463) = 7.5; *P* < .001]. Active/min did not differ significantly between weekday and weekend observation days [F(1,4464) = 0.45; *P* = .51] (Table 3).

**Table 3:** Descriptive statistics for active/min by subgroups.

	Estimated marginal means	Standard errors	Significance
Observation period			Morning < afternoon & evening (*P* < .001)
Morning	0.45	0.07	
Afternoon	0.85	0.08	
Evening	1.05	0.09	
City			Small > medium & large (*P* < .001)
West Chester (small)	1.21	0.13	
Wilmington (medium)	0.57	0.08	
Philadelphia (large)	0.49	0.11	
Observation day			No significant difference (*P* = .51)
Weekday	0.77	0.05	
Weekend	0.70	0.09	

**Fig. 3:**
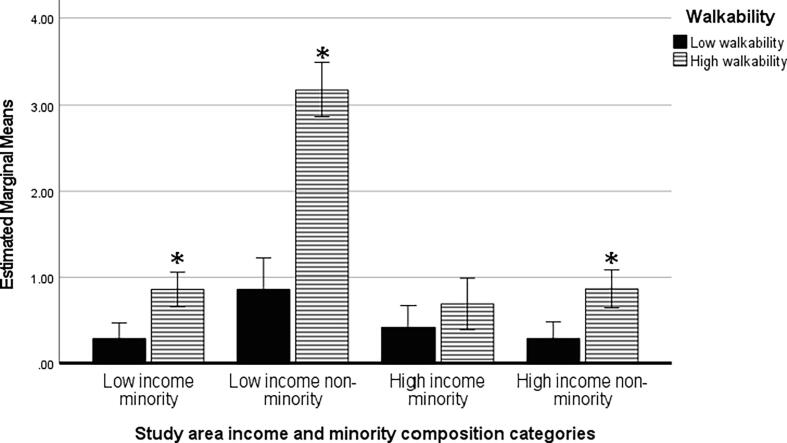
Active/min in low and high walkability areas by income and minority composition categories.

In the ANCOVA model, active/min was significantly associated with the interaction between walkability, income, and minority composition [F(1,4458) = 15.4; *P* < .001]. Follow-up analyses showed that simple effects were significant for walkability in low income minority areas [F(1,4458) = 17.0;*P* < .001: 0.86 +/- 0.1 active/min in high walkability areas > 0.29 +/- 0.09 active/min in low walkability areas], low income non-minority areas [F(1,4458) = 92.8; *P* < .001: 3.18 +/- 0.16 active/min in high walkability areas > 0.86 +/- 0.19 active/min in low walkability areas] and high income non-minority areas [F(1,4458) = 14.8; *P* < .001: 0.86 +/- 0.11 active/min in high walkability areas > 0.28 +/- 0.1 active/min in low walkability areas]. Simple effects for walkability were not significant in high income minority areas [F(1,4458) = 1.9; *P=*.17: 0.42 +/- 0.13 active/min in low walkability areas ∼ = 0.69 +/- 0.15 active/min in high walkability areas] (Fig. 3).

### 3.2 Comparative analyses non-active/min (controlling for meteorological conditions)

Non-PA/min did not differ by observation period [F(2,4463) = 1.4; *P* = .25]. More non-PA/min were observed in Wilmington (mid-sized) compared to Philadelphia (large-sized) [F(2,4463) = 4.3; *P* = .013]. Non-PA/min [F(1,4464) = 18.5; *P* < .001] was higher on weekend vs. weekday observation days (Table 4).

**Table 4:** Descriptive statistics for non-PA/min by subgroups.

	Estimated marginal means	Standard errors	Significance
Observation period			No significant difference (*P* = .025)
Morning	0.22	0.05	
Afternoon	0.24	0.05	
Evening	0.35	0.06	
City			Medium > large (*P* = .012)
West Chester (small)	0.28	0.08	
Wilmington (medium)	0.33	0.05	
Philadelphia (large)	0.08	0.07	
Observation day			Weekend > weekday (*P* < .001)
Weekday	0.19	0.03	
Weekend	0.47	0.05	

**Fig. 4:**
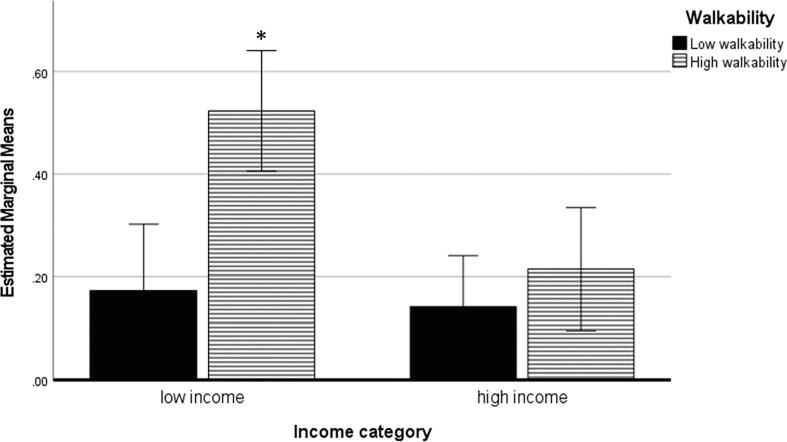
Non-PA/min in low and high walkability areas by income category.

In the ANCOVA model, non-active/min was significantly associated with the interactions between walkability and income [F(1,4458) = 5.10; *P* = .024] and walkability and minority composition [F(1,4458) = 6.63; *P* = .010]. Follow-up analyses showed that simple effects were significant for walkability in low income areas [F(1,4458) = 15.2; *P* < .001: 0.52 +/- 0.06 non-PA/min in high walkability areas > 0.17 +/- 0.07 non-PA/min in low walkability areas], but not significant in high income areas [F(1,4458) = 0.86; *P* = .36: 0.14 +/- 0.05 non-PA/min in high walkability areas ∼ = 0.22 +/- 0.06 non-PA/min in low walkability areas] (Fig. 4).

**Fig. 5:**
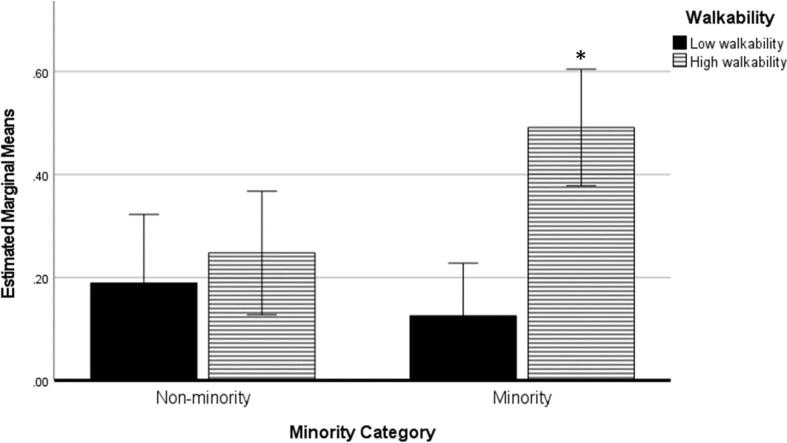
Non-PA/min in minority and non-minority areas by walkability.

For the walkability-minority composition interaction, simple effects were significant for walkability in minority areas [F(1,4458) = 23.1; *P* < .001: 0.49 +/- 0.06 non-PA/min in high walkability areas > 0.13 +/- 0.05 non-PA/min in low walkability areas], but not in non-minority areas [F(1,4458) = 0.42; *P* = .52: 0.19 +/- 0.05 non-PA/min in low walkability areas ∼ = 0.25 +/- 0.07 non-PA/min in high walkability areas] (Fig. 5).

## 4 Discussion

In this study, a relatively substantial number of objective observations were made of sidewalks/streets across a wide-range of circumstances using rigorous methodology. This supported the purpose of the study, which was to create a comprehensive description of PA and non-PA occurring on sidewalks/streets. The findings are valuable for providing a baseline or reference to which future evaluations of sidewalk/street use can be compared. They also offer insights into sidewalk/street use relative to walkability, socio-economic demographics, meteorological conditions, and other factors such as time of day.

The observation method has been used for decades to study human behavior (McKenzie et al., 2002). Within the last 20 years, it has gained prominence as a tool for describing PA in specific behavior settings such as school gyms, parks, and sidewalks/streets (Suminski et al., 2008, Suminski et al., 2006, McKenzie et al., 2002, Suminski et al., 2020, Suminski et al., 2019). While reports on PA in gyms and parks are numerous, little information has been published on PA and non-PA occurring on sidewalks/streets. Suminski et al (Suminski et al., 2008, Suminski et al., 2006) were the first to offer a detailed description of an observation method for counting people engaged in PA on sidewalks/streets along with contextual information. Since this time, their method has been employed to study relationships between PA, environmental factors, street renovations, and personal characteristics (Kelly et al., 2014, Suminski et al., 2008, Jensen et al., 2017, Jia and Fu, 2014). Beyond these studies, there has not been a larger-scale, descriptive study of sidewalk/street use even though this is a common behavior setting for both transport and recreational PA (Kang et al., 2017, Hurvitz et al., 2014, Suminski et al., 2015). In this regard, the current study has added importance in that it provides information on sidewalk/street use for PA and non-PA across a wide range of environmental circumstances, including meteorological conditions, that can serve as a baseline or reference to which outcomes from future evaluations of sidewalk/street use can be compared. The findings could also help guide assessments in this area by offering insight into what to look for, when to look for it, and where to look. This would be relevant for both researchers and practitioners alike.

Studies that have assessed PA on sidewalks/streets using observation report fairly similar results. For example, one study, focused on urban areas, found that most people using sidewalks/streets were walking (70%) and that on average, about one walker was seen/min of observation (Suminski et al., 2006). In suburban areas, sidewalk/street use is lower (∼0.5 walkers/min of observation) which would be expected, but as with urban areas, walking is the most prevalent behavior (Suminski et al., 2008). Other studies, using the same observation method, reported similar findings (Kelly et al., 2014, Jensen et al., 2017, Jia and Fu, 2014). The current study places the walk rate on sidewalks/streets around 1.6 walkers/min of observation and the prevalence of walking at 66.9% relative to all activities recorded. Clearly, walking is the most common PA performed on sidewalks/streets across an array of environmental conditions, the same conditions which appear to play a role in the use of sidewalks/streets for various activities. Fluctuations in both macro- (city) and micro-level (street segment) social/physical environmental factors coincide with sidewalk/street use (Kelly et al., 2014, Suminski et al., 2008, Suminski et al., 2008, Jensen et al., 2017, Jia and Fu, 2014). It is this type of experimental evidence that is needed to support and inform theoretical models that specifically identify aspects of the environment as important inhibitors/facilitators of PA (Sallis et al., 2006).

A primary strength of this study is evidence it provides about environment-PA/non-PA interactions operating within sidewalk/street settings. Less humid and windy conditions favored sidewalks/streets use for PA, while warmer, less humid and higher barometric pressures were associated with more sidewalks/streets non-PA. Similarly, a previous study reported significant correlations between meteorological conditions and cycling on urban streets and walking on an outdoor, oval track (Suminski et al., 2008). These findings suggest that when evaluating PA in outdoor settings, meteorological conditions should be factored in, especially when comparing areas or an area over time. The rate of sidewalk/street use for PA and non-PA also varied as a function of when observations were made. More active/min were seen in evenings and rates of non-PA were higher on weekends. While this study was not designed to explore reasons behind such a finding, it does support previous findings showing that sidewalk/street use expresses daily, temporal variations which may manifest as a function of activity type (Suminski et al., 2008, Suminski et al., 2013). For example, higher rates of PA during evenings might reflect an influx of recreational walkers, above normal walking for transport levels. Differences between cities in PA and non-PA were found, which is consistent with national data on U.S. cities (Centers for Disease Control and Prevention, 2020) and signifies the presence of macro-level phenomenon (e.g., infrastructure, culture) that operate in unison with micro-level factors (e.g., neighborhood walkability) to affect behavior (Centers for Disease Control and Prevention, 2020, Sallis et al., 2016, Thielman et al., 2015 Aug).

Walkability is consistently found to be a strong correlate of PA across multiple behavior settings as well as a good predictor of PA change over time (Thielman et al., 2015 Aug, Wasfi et al., 2017 Dec 19). In the current study, walkability was related to higher rates of sidewalk/street PA and non-PA, but these relationships were moderated by the income and minority composition of the study area. For instance, in high walkability areas, PA rates were 3.2 people/min in low income, non-minority areas, but only 0.9 people/min in high income, non-minority areas. Although such complexity has been found in previous studies, none of these studies utilized an objective measure of sidewalk/street use (particularly non-PA behavior) across wide ranges of environmental conditions (Kelly et al., 2007, Luijkx and Helbich,, Owen et al., 2018 Oct). Future research is warranted to identify the reasons income and minority status impact the effects of walkability on PA and non-PA and to the extent actual and perceived walkability play a role. This information might be vital for improving the effectiveness of certain efforts to promote PA (e.g., city-wide, walking campaigns).

Other strengths of this study include the use of a well-established, reliable, and valid method to assess sidewalk/street PA and non-PA (Suminski et al., 2006, Suminski et al., 2020, Suminski et al., 2019). The method incorporated video capture and analysis which has been shown to provide as good or better information on people in outdoor, behavior settings as traditional, low-tech approaches (29,30). The sample of sidewalk/street segments was taken from areas diverse with respect to the built environment (i.e., walkability), social characteristics (income, minority composition), macro-level factors associated with city size, and other dynamics related to when (time of day, day of the week) people were observed. Moreover, data were collected in 10 out of 12 months of the year, which resulted in true, “seasonal” meteorological variations and not just daily fluctuations in weather (e.g., cooler mornings to warmer afternoons). This distinction may be important for better understanding the relationship between seasonal PA changes and health outcomes in addition to the habitual nature of sidewalk/street PA and non-PA (Shephard and Aoyagi, 2009). In aggregate, the rigor and design of this study substantially enhance the usefulness of the results, especially with regards to applicability or generalizability to other areas.

Limitations also exist and should be considered when interpreting the results. Data collection were restricted to certain times of the day. This is consistent with time sampling methodology, which attempts to minimize the time needed for obtaining enough data to make generalizations about the larger phenomenon being assessed. Nevertheless, this is a limitation as it is possible other times of the day are relevant for knowing what is occurring overall. Possibly, 24/7 monitoring with surveillance cameras could overcome this issue. However, there are a number of problems associated with their use for studying human behavior that render this option unrealistic (La Vigne et al., 2011). Relatedly, time sampling is inherently inefficient. For instance, a considerable amount of time is devoted to observing behavior settings during periods of non-use. Suminski et al. (2022) reported that approximately 88% of park observation periods were void of people. Although this study implemented a complex data collection scheme to augment data diversity and promote generalizability, outliers undoubtedly exist to which study results would not be relevant. Nonetheless, most sidewalks/streets probably reflect a moderate amount of use (primarily walking) with a substantial period of time with little or no use. In addition, the observation routes were not randomly selected which could alter how well they represented the study areas. Most likely, this is a minor limitation given observation routes accounted for over 20% of the total linear feet of sidewalks/streets in a study area, the first segment of the route was randomly selected, and study areas were fairly homogeneous throughout regarding key outcomes (walkability, income) examined.

## 5 Conclusions

This study provides the first comprehensive report of observed PA and non-PA on sidewalks/streets across a wide range of environmental conditions. It demonstrates the utility of the observation method for assessing PA on sidewalks/streets where a considerable proportion of PA occurs. As such, the method could be used in public health research and practice to conduct place-based surveillance to examine factors affecting PA and/or detect changes in PA resulting from community-level interventions. The study also provides evidence supporting socio-ecological theories as applied to PA behavior, which posit that this behavior is in a dynamic relationship with several, interacting social and physical aspect of the environment. Therefore, results of this study have the potential to improve the effectiveness of PA promotion efforts. For instance, WalkScore could be utilized as a practical tool within lifestyle interventions to help practitioners and their patients recognize barriers and facilitators to using sidewalks/streets for recreational/transport PA. Finally, it will be critical to improve the efficiency of assessing sidewalk/street use in order to make such assessments feasible for large-scale applications. Most likely, this will involve employing machine learning/computer vision to automate data extraction from videos.

### CRediT authorship contribution statement

**Richard R. Suminski:** Formal analysis, Project administration. **Gregory M. Dominick:** Conceptualization, Methodology, Investigation, Writing - original & review, Visualization, Supervision, Funding acquisition.

## Declaration of Competing Interest

The authors declare that they have no known competing financial interests or personal relationships that could have appeared to influence the work reported in this paper.
